# High-resolution X-ray phase-contrast tomography of human placenta with different wavefront markers

**DOI:** 10.1038/s41598-025-85105-z

**Published:** 2025-01-16

**Authors:** Sara Savatović, Davis Laundon, Fabio De Marco, Mirko Riedel, Jörg U. Hammel, Madleen Busse, Murielle Salomé, Lorella Pascolo, Irene Zanette, Rohan M. Lewis, Julia Herzen, Pierre Thibault

**Affiliations:** 1 Munich Institute of Biomedical Engineering, Technical University of Munich, 85748 Garching, Germany; 2 Research Group Biomedical Imaging Physics, Department of Physics, TUM School of Natural Sciences, Technical University of Munich, 85748 Garching, Germany; 3 Chair of Biomedical Physics, Department of Physics, TUM School of Natural Sciences, Technical University of Munich, 85748 Garching, Germany; 4 Department of Physics, University of Trieste, 34127 Trieste, Italy; 5 Elettra - Sincrotrone Trieste S.C.p.A., 34149 Basovizza, Italy; 6 Human Development and Health, Faculty of Medicine, University of Southampton, Southampton, SO17 1BJ UK; 7 Institute for Life Sciences, University of Southampton, University Rd, Southampton, SO17 1BJ UK; 8 Institute of Materials Physics, Helmholtz-Zentrum hereon, 21502 Geesthacht, Germany; 9 ESRF - The European Synchrotron Radiation Facility, 38043 Grenoble, France; 10 Institute for Maternal and Child Health, IRCCS Burlo Garofolo, 34137 Trieste, Italy

**Keywords:** Image processing, X-rays, Biological fluorescence, Preclinical research

## Abstract

Phase-contrast micro-tomography ($\upmu$CT) with synchrotron radiation can aid in the differentiation of subtle density variations in weakly absorbing soft tissue specimens. Modulation-based imaging (MBI) extracts phase information from the distortion of reference patterns, generated by periodic or randomly structured wavefront markers (e.g., gratings or sandpaper). The two approaches have already found application for the virtual inspection of biological samples. Here, we perform high-resolution $\upmu$CT scans of an unstained human placenta specimen, using MBI with both a 2D grating and sandpaper as modulators, as well as conventional propagation-based imaging (PBI). The 3D virtual representation of placenta offers a valuable tool for analysing its intricate branching villous network and vascular structure, providing new insights into its complex architecture. Within this study, we assess reconstruction quality achieved with all three evaluated phase-contrast methods. Both MBI datasets are processed with the Unified Modulated Pattern Analysis (UMPA) model, a pattern-matching algorithm. In order to evaluate the benefits and suitability of MBI for virtual histology, we discuss how the complexities of the technique influence image quality and correlate the obtained volumes to 2D techniques, such as conventional histology and X-ray fluorescence (XRF) elemental maps.

## Introduction

Highly coherent synchrotron X-rays allow phase-sensitive imaging techniques to exploit refraction as a contrast mechanism for label-free samples. Phase information is widely used to increase contrast especially for low-absorbing biological materials by several orders of magnitude compared to the attenuation signal in the hard X-ray regime^1^. In combination with micro-tomography ($\upmu$CT), it gives access to high-contrast undistorted 3D data without damaging the sample. Unlike traditional histological methods that require slicing and staining, the sample can undergo further analysis with different techniques.

Phase-sensitive techniques such as grating-based imaging (GBI)^2,3^, edge illumination (EI)^4^ and speckle-based imaging (SBI)^5–7^ rely on analysing sample-induced changes to a pattern generated by a wavefront modulator. While GBI employs diffraction or absorption gratings to create interference patterns, EI is based on X-ray absorption within the masks and is independent of interference effects. In SBI, the wavefront modulator, often referred to as a diffuser, creates a random phase and absorption profile that produces intensity variations, i.e., speckles. The distinction between these methods lies in how they encode and disentangle the imaging signals within various experimental arrangements and reconstruction methods.

SBI methods typically rely on a pixel-wise correlation analysis or cost function minimisation to retrieve the information. Differences associated with changes in the speckle pattern caused by the sample can be modelled to extract absorption, phase and dark-field signals. The same analysis can be applied to a wavefront marked with a periodically structured illumination, such as one-dimensional gratings^8,9^ and two-dimensional gratings (i.e., grids, TAIs)^10–12^. This more generalised approach can then be referred to as the modulation-based imaging (MBI) technique.

Besides ‘X-ray Speckle-Vector Tracking’^13^ or ‘X-ray Multi-modal Intrinsic-Speckle-Tracking’^14–16^, and others^17–19^, ‘Unified Modulated Pattern Analysis’ (UMPA) is a pattern-tracking algorithm capable of processing such datasets^20,21^. UMPA enables the simultaneous retrieval of the transmission, orthogonal differential phase shifts, and small-angle scattering signals.

In this study, we focus on examining image quality of the reconstructed phase volumes obtained with MBI using synchrotron radiation and wavefront markers of varying structural complexity, namely Talbot array illuminators (TAI) and sandpaper. In addition, we investigate how the reconstructions compare to the propagation-based imaging (PBI) method. While MBI is sensitive to phase gradients, PBI is sensitive to the Laplacian of the phase and it makes use of contrast enhancement effects at interfaces that occur when increasing the distance from sample to the detector. The phase signal is generated from the interference between differently shifted wave fronts, which gives rise to fringes along the edges of the sample features. Phase retrieval in PBI does not require additional optical elements and relies on a low-pass filter proposed in Ref.^22^. Given its simple experimental implementation and fast processing, it is well suited for fast tomographic acquisitions. The method’s application is, however, constrained by sample composition assumptions^22^ and the setup’s spatial coherence^23^, constraints that are less relevant in MBI^24,25^.

Recently, tomographic MBI datasets processed with the faster, re-implemented version of UMPA in C++^21^, have demonstrated the ability to provide high-resolution virtual histology of biological specimens with both sandpaper^26^ and TAIs^27^, granting access to perfectly co-registered multi-modal information, including insights on the sample’s unresolved microstructure through dark-field^28^.

Here, we investigate human placental villous tissue at high spatial resolution to examine micrometre-sized morphological features in three dimensions, accessible with phase-contrast $\upmu$CT. We then correlate the results with conventional histology and X-ray fluorescence (XRF) microscopy to accurately interpret the virtual images within a biological context.

The mammalian placenta is a structurally diverse organ^29^, important for ensuring the fetus’ wellbeing and proper development. It is a unique organ with several vital physiological functions that ensure proper delivery of oxygen and nutrients to the fetus, while also providing defense and detoxification from toxins and metabolites. As a result, a quantitative 3D virtual representation of the villous structure organisation of the human placenta can aid in a better understanding of its function and potential effects on fetal health^30^. Moreover, it facilitates a clearer identification of relationships between various structures within the densely-packed tissue.

## Materials and methods

### Sample preparation

Term placental tissue was collected after delivery from an uncomplicated pregnancy with written informed consent. Ethical approval for this study was obtained from the Southampton and Southwest Hampshire Local Ethics Committee (11/SC/0529). All the methods in the study were performed in accordance with the relevant guidelines and regulations. The placental tissue was dissected and fixed in neutral buffered formalin overnight before being transferred to PBS. A smaller piece of the placental tissue was later cut into smaller portions at the Munich Institute of Biomedical Engineering (Technical University of Munich, Germany), and processed using a Shandon Excelsior ES automatic tissue processor (Thermo Fisher Scientific, U.S.). The tissue was embedded into a paraffin block after an ethanol-dehydration series. The block was cut into a cylindrical shape and attached to a sample holder for the tomographic measurements. The final size of the sample was about $4.5 \times 4.5 \times 3\,\hbox {mm}^{3}$. A bigger sample from the same batch was recently analysed in Ref.^31^.

### Experimental setup

**Fig. 1:**
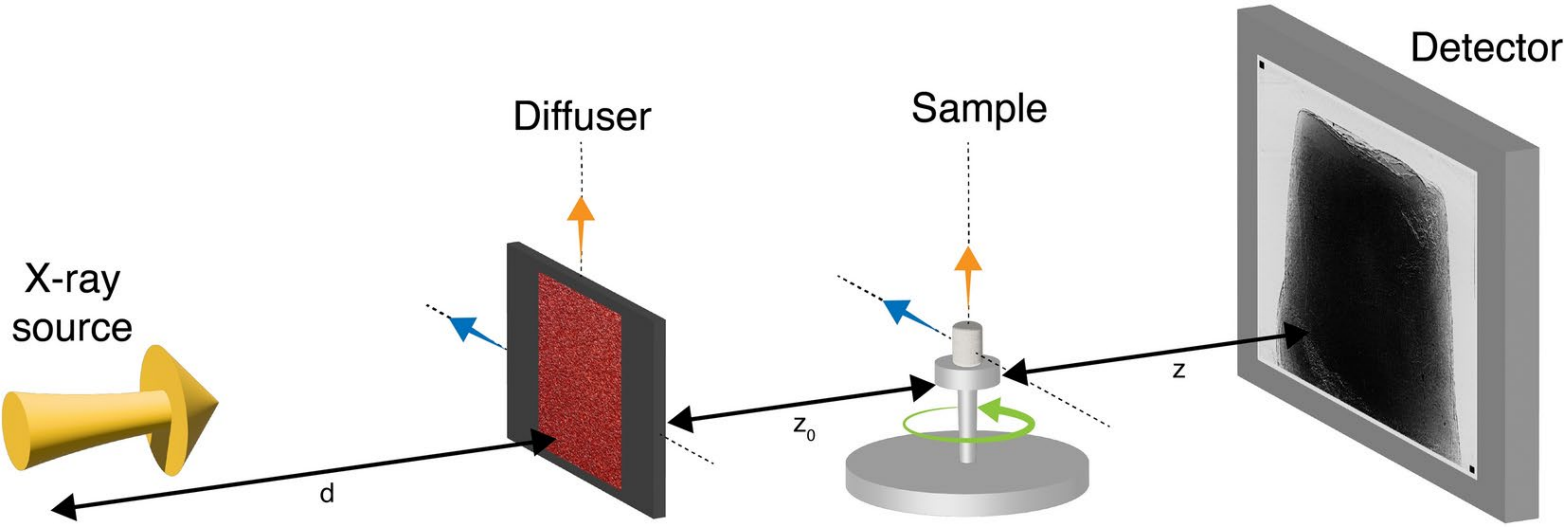
Experimental setup at the imaging beamline P05, PETRA III (DESY, Hamburg, Germany). The modulator was mounted on translation stages for lateral translation.

The measurements were performed at the imaging beamline P05 operated by the Helmholtz–Zentrum Hereon at PETRA III (DESY, Germany). A double-crystal silicon monochromator was used to produce 20 keV X-rays from an undulator insertion device located 85 m from the micro-tomography end-station^32,33^. The images were acquired with a custom-built CMOS camera^34^ optically coupled to a ${100}\,\upmu \hbox {m}$ cadmium tungstate ($\hbox {CdWO}_4$) scintillator. We used a $5\times$ magnification, which resulted in a sensitive area of ${6.55 \times 2.75}\,\hbox {mm}^{2} \,({5120 \times 2151} \,\hbox {pixel}$) with an effective pixel size of ${1.28}\,\upmu \hbox {m}$ in the sample plane. All the images were taken with an exposure time of 80 ms. The system resolution was estimated from a slanted edge modulation transfer function, resulting in ${2.14}\,\upmu \hbox {m}$ (1.67 pixel). A schematic of the main components of the experimental setup is shown in Fig. 1. It includes a modulator, mounted on a two-axis motorised translation stage, in addition to a standard tomographic setup.

### Multi-modal tomography with MBI

**Fig. 2:**
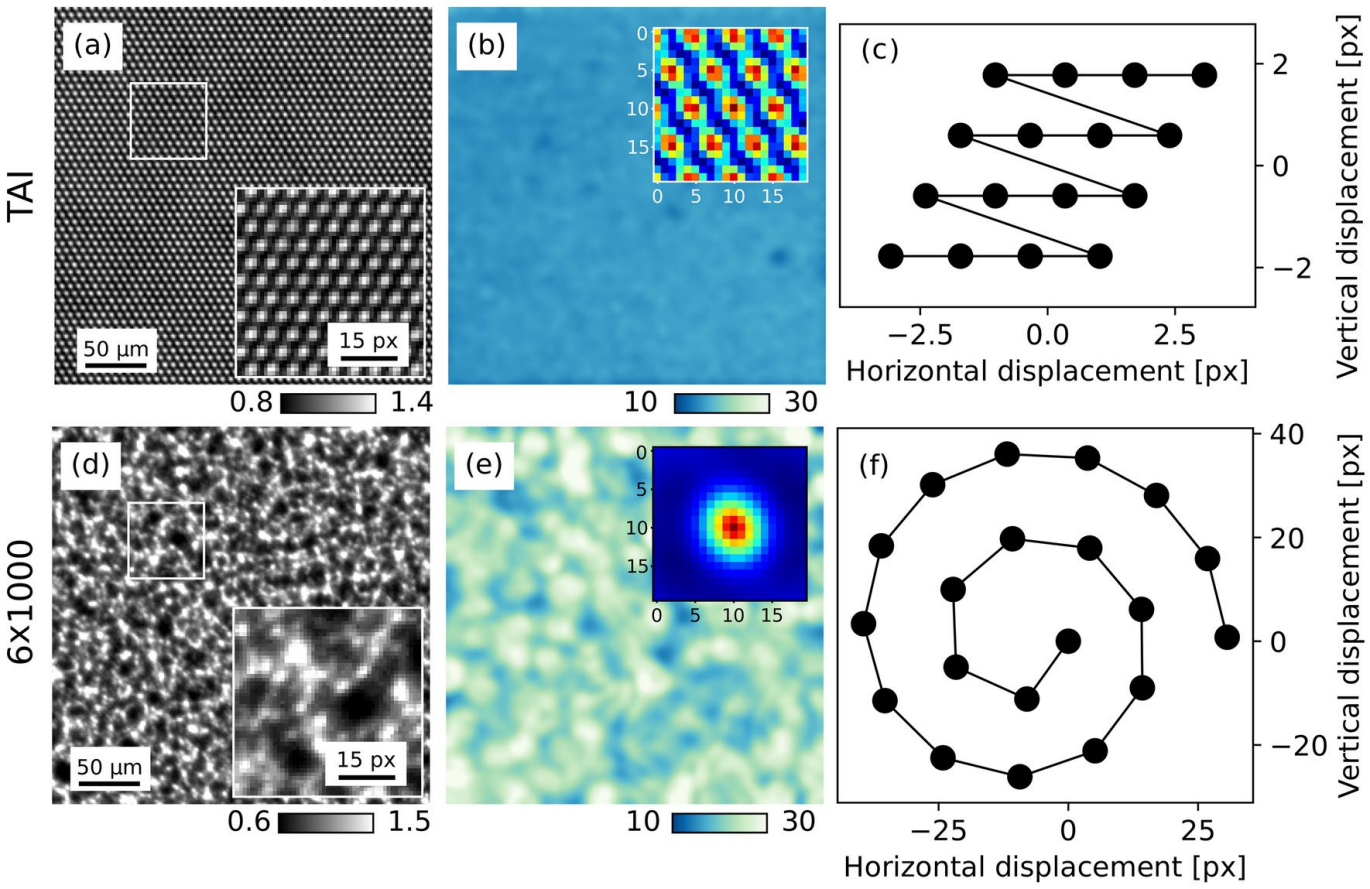
Modulator reference maps, visibility analysis and stepping patterns for the two wavefront markers. **(a)** Reference image details from the centre of the FOV of the ${7}\,\upmu \hbox {m}$ period TAI and **(d)** the ${6 \times 1000}$ grit sandpaper, with zoomed insets of the highlighted region. **(b,e)** Show the visibility maps and the insets show the speckle pattern 2D auto-correlation map (${20 \times 20}$ pixel around the peak—dark red, base—blue). The insets aid in the visualisation of the mean size of the marking structures ($\textrm{FWHM}_{\textrm{TAI}}$ = 2.85 pixel vs. $\textrm{FWHM}_{\textrm{6x1000}}$ = 5.82 pixel). The estimated mean visibilities for the TAI and sandpaper are 15.0% and 22.5%, respectively. The modulator stepping patterns for the two acquisitions are shown in **(c,f)**.

For MBI tomography, we used ${7}\,\upmu \hbox {m}$ period hexagonal silicon TAI and 6 layers of 1000 grit silicon carbide sandpaper (‘${6 \times 1000}$’ in the following) as wavefront markers. The modulators were motorised and stepped in the plane transverse to the beam direction following the patterns shown in Fig. 2c,f: a tilted grid for the TAI to scan one unit cell (16 steps) and a spiral for the sandpaper (20 steps). For each modulator step, 3001 angular views of the sample were acquired in a continuous ${180}^{\circ }$ tomographic scan. 20 dark images and 70 reference images were taken before and after each tomographic scan. The raw images were dark current and beam profile corrected. Bad pixel outliers were replaced by the median of their closest neighbours; the beam profile was estimated from the low-pass filtered (50 pixel kernel size) reference images. The set of sample and reference frames recorded at different modulator positions were processed using UMPA^21^ with a ${3 \times 3}$ analysis window for each tomography angle after modulator drift corrections^35^. Within small-angle approximation, the resulting differential phase signals $(\frac{\partial \Phi }{\partial x}$, $\frac{\partial \Phi }{\partial y})$ along the two orthogonal directions are proportional to the pixel displacements provided by UMPA ($u_{x}$, $u_{y}$). Absolute phase shifts $\Phi$ induced by the object were obtained by integrating the two orthogonal channels with the Fourier method described in Ref.^36^. A 2D polynomial of first order was fitted to the background areas of the differential signals and subtracted before integration. Subsequently, a second order polynomial was fitted to the integrated phase, and subtracted to reduce residual low frequencies. The tomographic reconstructions were obtained with a conventional filtered back-projection from ASTRA Toolbox^37,38^, after applying a Butterworth band-pass filter to the sinogram for ring artifact corrections. The reconstructed quantity is the decrement $\delta$ of the real part of the sample’s complex refractive index $n = 1 - \delta + i\beta$, which can be converted to electron density $\rho _{e}$ using

1 $$\begin{aligned} \rho _{e} = \delta \frac{k^{2}}{2 \pi r_{0}}, \end{aligned}$$

where $r_{0}$ is the classical electron radius and $k = 2\pi /\lambda$ is the wave number. In the same way, the real part of the refractive index $\beta$, related to the linear attenuation coefficient $\mu$^39^, is obtained from the transmission projections retrieved with UMPA.

### Phase contrast tomography with PBI

The modulator was moved out of the beam for the single-distance PBI measurements. Given the experimental parameters in Table 1, the sample was placed 93 mm from the detector, within the recommended distance range for single-distance propagation phase contrast tomography^40^. The acquired images were corrected for the dark current and the mean beam profile calculated from 70 frames before and after the tomographic acquisition prior to applying the phase retrieval filter^22^. A phase-retrieval parameter $\gamma = \delta / \beta = 202.43$ was chosen from tabulated values of soft tissue in wax (*xraylib*^41^). However, the value of $\gamma$ can be experimentally tuned to optimise the image appearance.

**Table 1:** $\upmu$CT experimental setup parameters.

Energy (keV)	20
Pixel size ($\upmu \hbox {m}$)	1.28
z (PBI) (mm)	93
z (MBI) (mm)	175
z$_{0}$ (mm)	115
d (m)	85
Angles	3001
Acquisition time per frame (ms)	80

### Histology

Conventional histology was performed on the same sample a few months after the tomographic measurement. The sample was re-embedded in paraffin, attached to a histological cassette, and sent to Morphisto Ltd., Germany for slicing and staining. Five different levels of the sample were taken (sections every ${100}\,\upmu \hbox {m}$, ${5}\,\upmu \hbox {m}$ thick). The sections were dewaxed, rehydrated and stained using standard haematoxylin and eosin (H&E) and periodic acid-Schiff (PAS) protocols. The sections were then imaged using a Zeiss Axioskop plus microscope equipped with a built-in camera AxioCam MRc (Carl Zeiss AG, Germany). The pixel sizes in the sample plane were ${1.33}\,\upmu \hbox {m}$ and ${0.66}\,\upmu \hbox {m}$ for the $\times 5$ and $\times 10$ objectives, respectively.

### XRF imaging

2D $\upmu$XRF measurements were performed at the ID21 beamline at the European Synchrotron Radiation Facility (ESRF, France). The sample was scanned at 7.3 keV photon energy (Si(311) monochromator–0.15 eV resolution) with a step size of ${3}\,\upmu \hbox {m}$ for the low resolution and ${0.5}\,\upmu \hbox {m}$ for the high resolution maps, respectively. A micro-beam of size ${0.3}\,\upmu \hbox {m} \times {0.8}\,\upmu \hbox {m}$ (V x H) at the focus spot, incident at ${62}^{\circ }$ to the sample plane^42^, was delivered by Kirkpatrick-Baez mirrors. Similar works^43^, report on delivering, point by point, an estimated dose of the order of $1 \times 10^{9}\,\hbox {Gy}$. A single-shot detector, located at an angle of ${90}^{\circ }$ from the incoming beam, collects the XRF photons point by point in a raster scan, with an acquisition time of 200 ms per pixel. The acquired spectra were then processed and fitted by PyMCA, a software using least-squares fitting algorithms and SNIP background subtraction routine, producing XRF elemental distribution maps. This ensured that the background noise was removed and that the remaining peaks were statistically relevant. The scanned slice was ${5}\,\upmu \hbox {m}$ thick and no staining was used. During the XRF scan, the slice was positioned on a ${4}\,\upmu \hbox {m}$ thick sheet of ultralene film and secured within a circular sample holder.

## Results

### Wavefront marker characterisation

Here, we examine the phase volume reconstruction from MBI with sandpaper and TAIs, measured within the same experimental arrangement. The method’s performance at an imaging system is usually characterised by the reference pattern visibility^24^. In MBI with sandpaper, the pattern’s modulation is due to interference effects and changes with sandpaper grain size, composition, and propagation distance^44^. For the TAIs it can be affected by composition, cell size, etching depth, and propagation distance^11^. The phase information can be extracted from a single exposure with UMPA; however, scanning the modulator improves phase sensitivity and spatial resolution by incorporating complementary information from the different steps. The stepping pattern was changed between the two acquisitions to optimise the pattern sampling over the field-of-view (FOV)^11,24^ (see Fig. 2c,f).

Visibility is defined as the local standard deviation over the mean value of the reference image, for each pixel^45^. We adopt the same definition for our analysis on both MBI datasets. Figure 2a,d show a ${250 \times 250}$ pixel region of interest (ROI) in the central part of the wavefront markers in the FOV; they highlight the differences in the marking structures arrangement. The hexagonal TAI exhibits a homogeneous visibility throughout the FOV, with a mean value of 15%, whereas the sandpaper generates a random speckle pattern with a visibility of 22.5%. The latter has a mean speckle size of 5.82 pixel (${7.45}\,\upmu \hbox {m}$), estimated as the full width at half maximum (FWHM) of the pattern’s 2D auto-correlation function^24^. The same analysis was performed on the TAI pattern, indicating a $\textrm{FWHM}_{\textrm{TAI}}$ of 2.85 pixel (${3.65}\,\upmu \hbox {m}$) and thus a finer sampling. The measured values are in accordance with the specifications of the wavefront markers when taking into account the imaging system point spread function. The silicon carbide sandpaper was composed of particles with a mean size of ${5.8}\,\upmu \hbox {m}$. The ${7}\,\upmu \hbox {m}$ hexagonal lattice etched in ${200}\,\upmu \hbox {m}$ thick silicon wafers had a duty cycle of 1/3, etching depth of ${17}\,\upmu \hbox {m}$, and a phase shift of $2\pi /3$ for the selected energy (5 microns, Germany). The 2D auto-correlation functions for both patterns are shown in the insets of Fig. 2b,e.

### Phase-contrast tomography

**Fig. 3:**
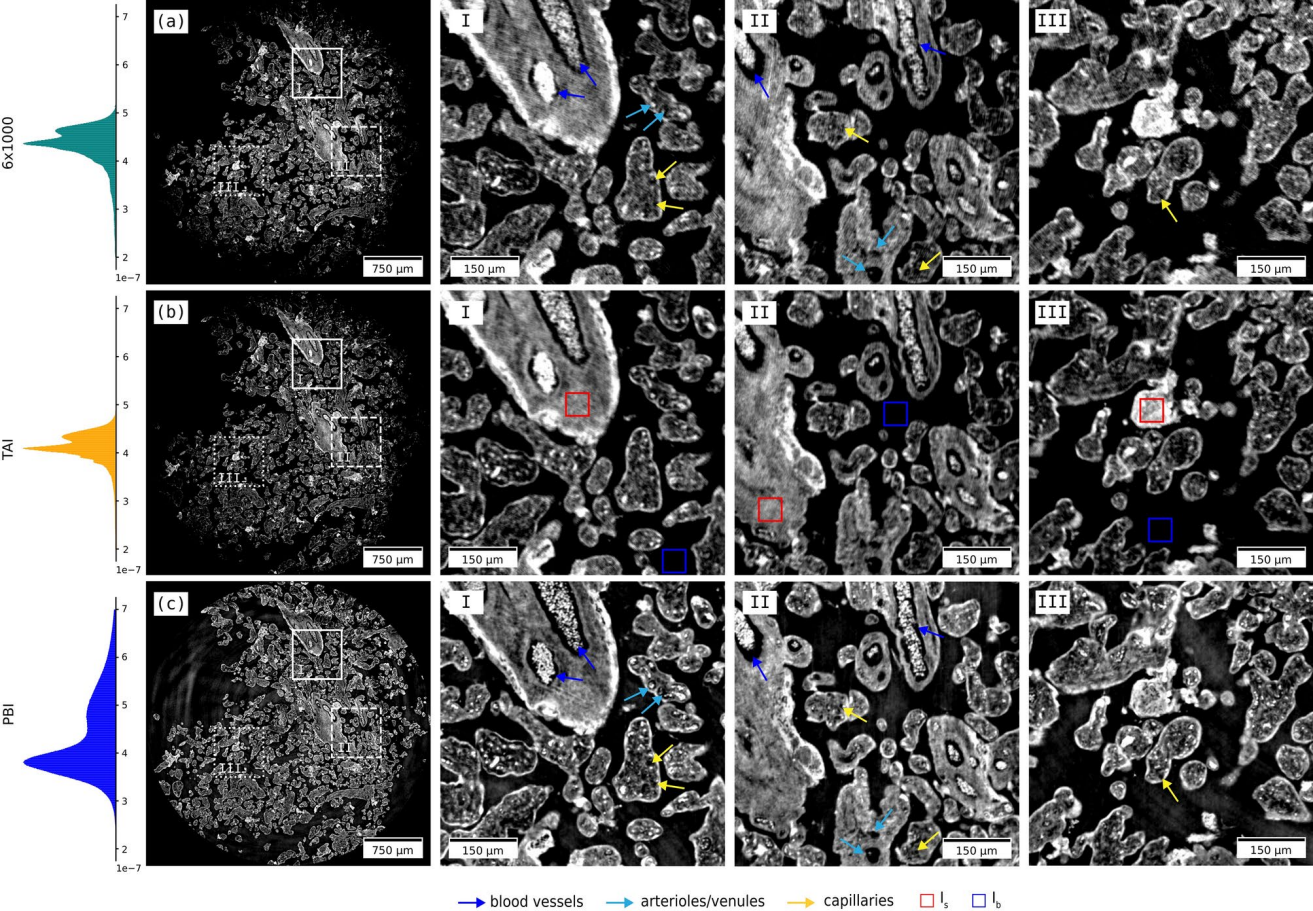
Same axial slice of the placenta sample’s tomographic phase reconstruction with the three examined measurement techniques: **(a)** MBI with sandpaper, **(b)** MBI with TAI, and **(c)** PBI. The volumes are aligned and the zoomed ROIs (**I**–**III**) on the right show identical sample details. The red and blue boxes indicate the sample and background ROIs (${40 \times 40}$ pixel) used for estimating image quality parameters.

Axial slices at the same height in the three phase-contrast volumes are shown in Fig. 3 with zoomed insets of the same ROIs. Visually, the details within the three reconstructions exhibit comparable image quality.

MBI phase-contrast volumes were obtained by processing the datasets with different wavefront markers using UMPA. Ring artifacts were more pronounced for the measurements using sandpaper, requiring an adjustment of the Butterworth filter settings, compared to the TAI volume. This could be due to the coarser sampling of the wavefront and larger wavefront marking structures, even though a higher number of modulator steps were acquired for the sandpaper dataset. This suggests that the visibility and number of modulator steps are not always parameters that determine the final performance of MBI tomography for a given setup. The speckle size, or wavefront marking structure size, can also greatly influence the MBI phase retrieval at high-resolution setups. Moreover, at high spatial resolution, the tracking algorithm can be affected by the slightest system vibrations, which can result in reconstruction artifacts and influence the differential phase signal retrieval. The $\upmu$CT hutch EH2 at P05 features a massive granite base stage mounted on a tripod, which supports all motors, along with the sample and detection systems which are mounted on tripods themselves. This design minimizes potential external environmental sources of vibration and is able to move with ${1}\,\upmu \hbox {m}$ precision allowing for very precise alignment^33^. Despite this, beam fluctuations remain a challenge. Given that the monochromator is located 85 m away from the detector, even minor fluctuations can amplify imperfections present on elements in the X-ray path, impacting the beam profile stability and overall pattern-matching system performance. These system imperfections can be corrected by means of drift correction functions^35^ or principal component analysis (PCA) methods^46^ to synthesise an optimal reference image that matches the sample’s pattern^27^. The latter, however, requires an empty background ROI in the sample image for the synthesis.

The PBI volume, on the other hand, exhibits good spatial resolution but suffers from low-frequency artifacts from beam imperfections and reveals inhomogeneities in uniform parts of the sample. The principal limitation concerning single-distance free-space phase-contrast imaging is that it provides qualitative information, and does not distinguish between the object’s phase and absorption contributions. The absence of this information prevents the calculation of the exact density of the material or possibly retrieving its chemical composition. Unlike single-distance PBI, the multi-distance approach requires greater experimental and computational complexity. It involves scanning the sample at multiple propagation distances and applying contrast transfer function-based signal extraction methods^47^. The sample has to be positioned precisely to avoid misalignments across the distances. In turn, this approach provides both phase and attenuation signals. However, neither free-space propagation method is suitable for investigating optically thick or mixed objects composed of arbitrary absorbing and phase-shifting components, a limitation that does not apply to MBI^27^.

### Image quality

Image quality parameters such as spatial resolution, phase sensitivity, contrast-to-noise ratio (CNR), and the Natural Image Quality Evaluator (NIQE) are useful in understanding the differences between phase-contrast tomographic reconstructions from different techniques. Here, we evaluate the spatial resolution with a method that considers a global noise level threshold as resolution limit in the Fourier power spectrum of the image. The noise level is estimated from the standard deviation in a homogeneous ROI of the volume, a method similar to the one in Ref.^48^, covered in more detail in Ref.^28^. The *spatial resolution* measurements in the three volumes indicate that PBI performs better than MBI, with a measured value of ${5.1}\,\upmu \hbox {m}$, in contrast to the ${8.2}\,\upmu \hbox {m}$ and ${7.7}\,\upmu \hbox {m}$ for the TAI and sandpaper SBI datasets, respectively. *Angular sensitivity* is a quality parameter which describes the sensitivity of the differential phase signal measurement. It is defined as the standard deviation in a homogeneous background ROI of the horizontal and vertical refraction angle image outside the sample. Here we report mean values of about 318 nrad and 399 nrad for the TAI and sandpaper datasets, respectively. In phase-contrast tomography, the projection angular sensitivity is reflected to the volumetric reconstruction and influences electron density resolution, i.e. *phase sensitivity*—the smallest resolvable electron density difference. The TAI demonstrates greater angular and phase sensitivity compared to sandpaper, likely due to the finer marking structures. *CNR* evaluates the signal level in the presence of noise. In our case it was estimated by considering the red and blue boxes in Fig. 3b as signal and background ROIs for the mean value ($I_{s}$, $I_{b}$) and standard deviation $(\sigma _{b}$) measurements. The results indicate that both MBI datasets perform comparably well in terms of contrast, slightly better than the PBI dataset. The histograms in Fig. 3 indicate that the MBI datasets have narrower $\delta$ value distributions. The *NIQE* is a blind image quality analyser that only makes use of measurable deviations from statistical regularities observed in natural images^49^. A smaller number suggests an image with a more natural look to human perception. In our case, the values indicate very similar results, with the TAI dataset achieving a slight advantage. All the abovementioned quality parameters are reported in Table 2. By assessing these image quality parameters, it is possible to systematically evaluate the performance of different approaches.

It should be noted that these results can change depending on the parameters used for the phase retrieval. PBI can be tuned by changing the phase-retrieval parameter $\gamma$, however, values close to the theoretical sample composition should be used when available^50^. In the same way, MBI reconstructions will yield different values when modifying the analysis window size^19,20^. In this work we selected reconstruction parameters in such a way to optimise spatial resolution. This means we chose a small analysis window in UMPA and selected a tabulated $\gamma$ that doesn’t filter excessively but still reduces most of the edge enhancement fringes at tissue interfaces.

**Table 2:** Estimated image quality parameters for the different phase-contrast reconstructions.

Method	Resolution ($\upmu \hbox {m}$)	$\sigma _{\alpha _x}$ (nrad)	$\sigma _{\alpha _y}$(nrad)	$\sigma _{\rho _e}$(#electrons/Å^3^)	CNR	NIQE
MBI (TAI)	8.2	331.2	306.4	0.00152	$6.3\pm 1.3$	20.41
MBI (6x1000)	7.7	428.2	369.0	0.00206	$5.8 \pm 1.1$	20.44
PBI	5.1	n.a.	n.a.	0.00890	$3.0 \pm 0.7$	20.59

### 3D virtual histology of human placenta

In the previous section we reported and discussed the image quality for the phase reconstructions. Considering the comparable obtained results, we opted to use the classic MBI with sandpaper, to demonstrate how virtual histology can contribute to a biological application.

**Fig. 4:**
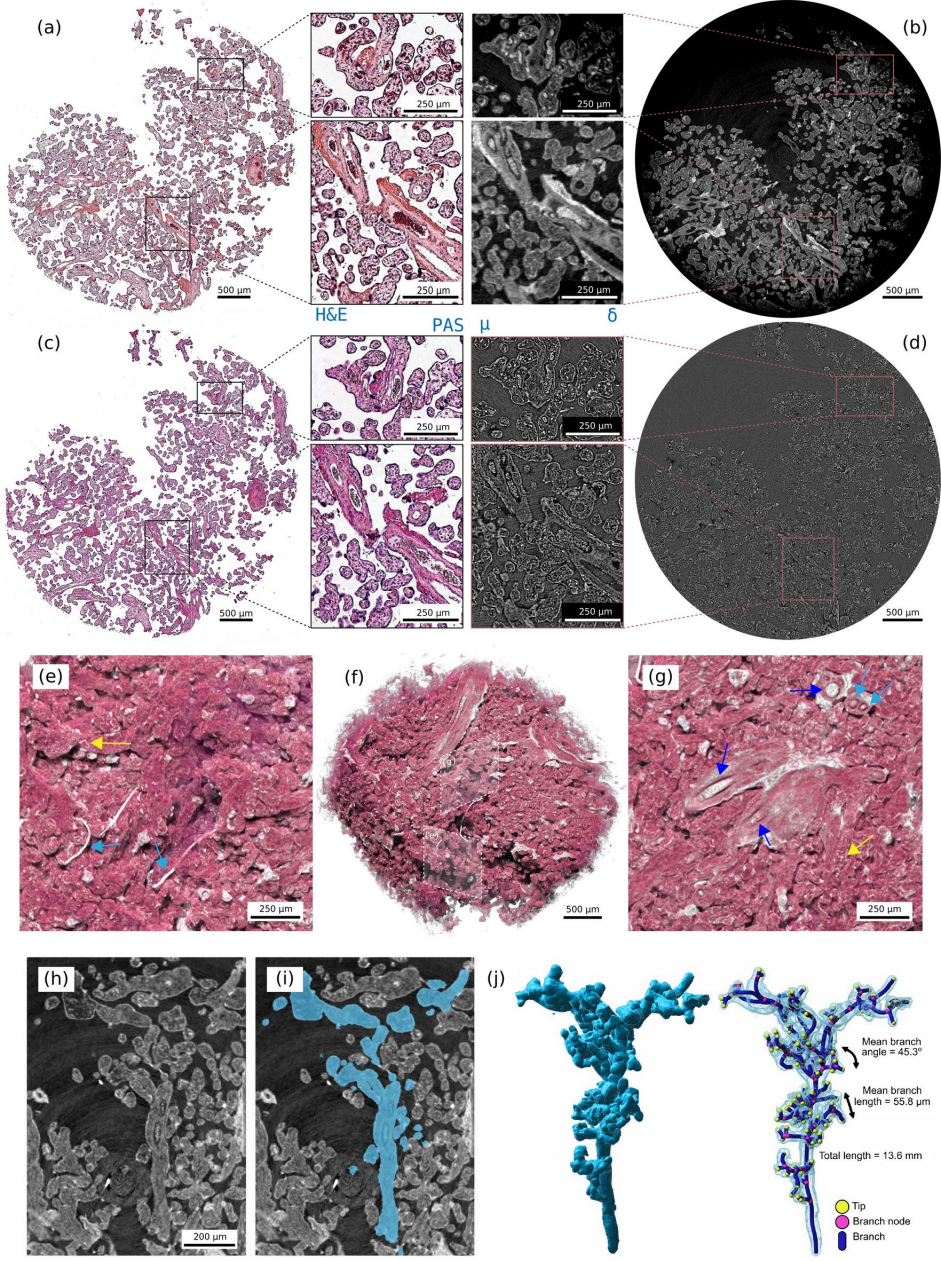
**(a)** H&E and **(c)** PAS-stained histology sections with the corresponding reconstructed **(b)** phase and **(d)** attenuation volume slices in the MBI sandpaper volume. **(f)** Rendering of the phase volume with different views in the volume in **(e,g)**. Dark and light blue arrows indicate bigger and smaller blood vessels, respectively. **(h–i)** Show a slice in the phase volume along with the segmentation of a single villous tree. **(j)** Reports its 3D representation and branching analysis.

Figure 3 shows an axial slice of the phase volume with some labels: major blood vessels (blue arrows), arterioles and venules (light blue arrows), and capillaries (yellow arrows). Figure 4 reports a more comprehensive visualisation of the datasets. Panels (a)–(d) show a comparison between $\upmu$CT and conventional histology. $\upmu$CT datasets were resliced to correlate the XY orientation to the histology block face using Avizo (ThermoScientific) as in Ref.^51^. Cross-sections of the villi and surrounding tissue are shown for H&E and PAS-stained histology slices in Fig. 4a,c. The correlated slice in the phase volume is shown in Fig. 4b and attenuation in Fig. 4d. Fibrin deposits are thought to occur where there is damage to the syncytiotrophoblast and are accentuated around the vessels and easily visible in the phase contrast volume. Different structures can be identified: the syncytiotrophoblast envelope surrounding the villous section, blood vessels, and connective tissue (stroma)^52^. Single blood cells (${\sim }6\,\upmu \hbox {m}$) can be distinguished in the circulatory system sections. Figure 4e–g show renderings of the phase volume and highlight the three-dimensional spatial arrangement of the villi. Blue arrows point to major blood vessels. In order to confidently segment a single villous tree in the densely-packed 3D villi network, some manual selection was necessary. The high contrast of the villous structures with paraffin wax facilitated this task. A villus branching tree was segmented from the phase-contrast $\upmu$CT dataset using Microscopy Image Browser^53^, as performed in Ref.^51^. The segmentation label was imported into Imaris (Oxford Instruments, U. K.) and its branching morphometry quantified using the ‘filament tracer’ tool. Figure 4h–j show how the single tree distributes in a two-dimensional slice within the volume. This highlights the challenge of tracing and mapping parts of a single villus using traditional two-dimensional techniques. The ability to virtually extend the information to three dimensions with phase-contrast $\upmu$CT overcomes this limitation and provides equivalent information. Figure 4j shows the segmented villous, the structure had a total length of 13.6 mm, with a mean branch length of ${55.8}\,\upmu \hbox {m}$ and mean branching angle of ${45.3}^{\circ }$.

### Correlation of virtual histology to XRF maps

**Fig. 5:**
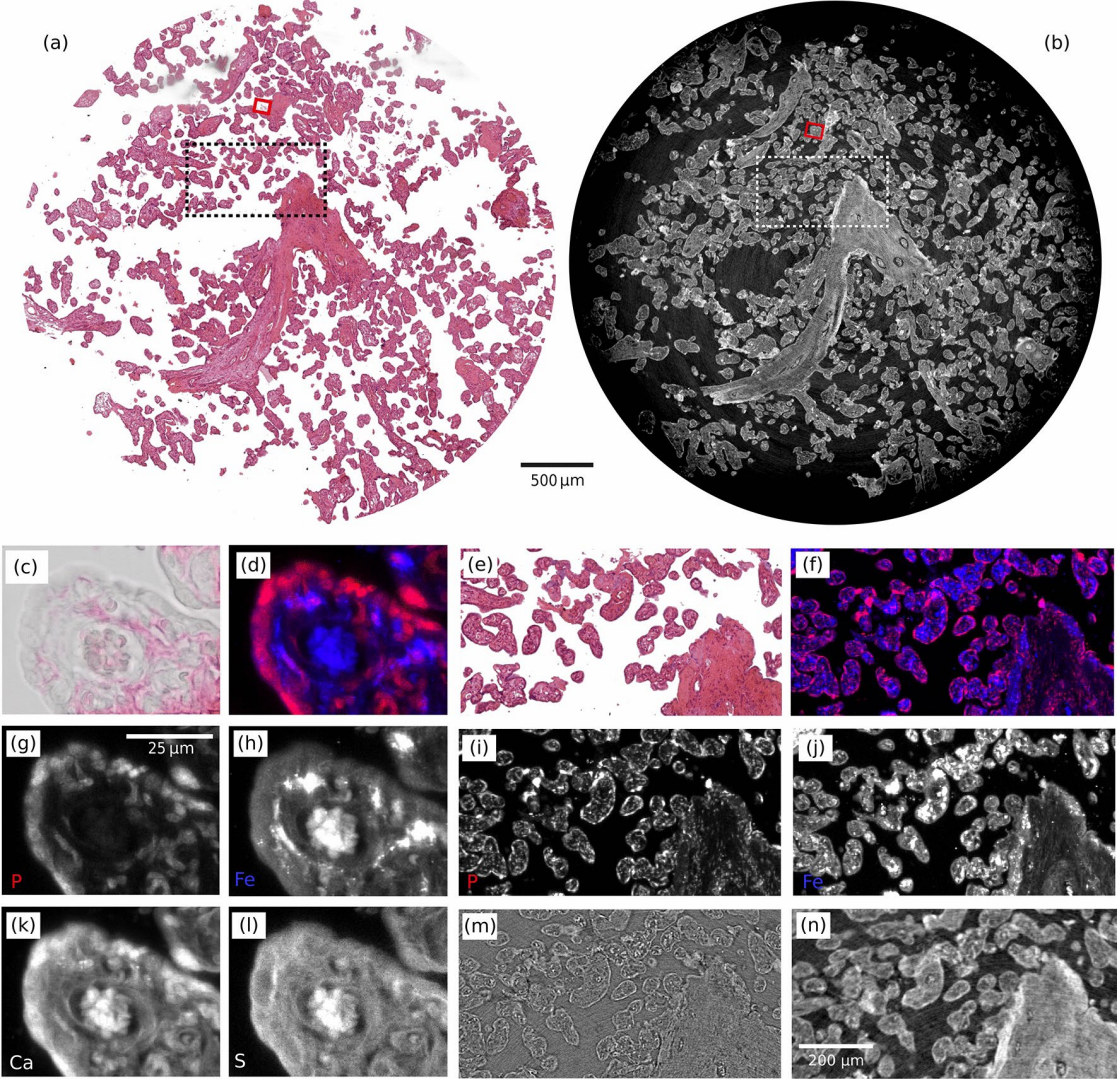
Correlation between X-ray phase contrast tomography, conventional histology, and 2D $\upmu$XRF maps. **(a)** Conventional histology slice stained with H&E. **(b)** Same slice in the phase-contrast volume. The red boxes in **(a,b)** indicate the field of view (FOV) of the high-resolution XRF scan, while the dotted boxes represent the FOV of the lower resolution XRF scan. **(c)** Zoomed ROI delimited by the red box in **(a)** indicates that H&E staining was not successful (faded colouring), because the tissue properties were changed by radiation damage during the XRF scan. **(d)** Overlay of phosphorous (P) and iron (Fe) elemental maps, shown in red and blue, respectively, with individual maps displayed in panels **(g,h)**. Additionally, we report calcium (Ca) and sulphur (S) elemental maps in panels **(k,l)**. Elemental maps are normalized, with black corresponding to the weakest and white to the strongest XRF signal. The high-resolution XRF scan has $3\times$ higher spatial resolution, making it challenging to compare these details directly with the X-ray phase-contrast scan. In contrast, the lower-resolution XRF scan has a comparable resolution to virtual histology. **(e)** Shows the ROI delimited by the black dotted box in conventional histology in **(a)**. **(f)** XRF elemental map overlay for the lower-resolution scan (P and Fe maps are shown below in **(i,j)**). **(m,n)** Show the same detail in the attenuation and phase-contrast volumes (white dotted box in **(b)**).

A $5\,\upmu$m thick unstained slice of the same sample was scanned at the X-ray microscopy beamline ID21 (ESRF, France) to retrieve XRF 2D elemental maps at two spatial resolutions, with ${3}\,\upmu \hbox {m}$ and ${0.5}\,\upmu \hbox {m}$ scanning steps on ${270 \times 140}$ and ${180 \times 140}$ matrices, respectively. Spectra were acquired for each raster scan step and the different elemental peaks calibrated to obtain the elemental distribution maps. After the scan, the slice was sent to collaborators at the Institute for Life Sciences, University of Southampton, United Kingdom for conventional histology. The slice was transferred from ultralene film to a glass slide and stained with H&E. This procedure proved challenging, and it may have introduced some deformations to the original sample shape, visible from the comparison of Fig. 5e.

Figure 5 shows the correlated images of the XRF scans at two resolutions, conventional histology and virtual histology. Image correlation was performed following the same steps described in the previous section. The nuclei within the syncytiotrophoblast are highlighted in the phosphorus elemental map, on the outer edge of the villi in Fig. 5d, while stromal cells are visible in the connective tissue where phosphorus and iron elemental map overlap (purple pixels in (d)). Interestingly, iron accumulates in stromal cells and as expected in erythrocytes (inside blood vessels, central region in (d)), but not in the syncytiotrophoblast. In addition, we show the calcium and sulphur elemental maps for the higher-resolution XRF scan. The calcium map highlights the morphology of the sample, while the sulphur map emphasises the supportive connective tissue, the villous stroma^52^.

## Discussion and conclusion

The human placenta proved to be a suitable sample for this study because it has an intricate three-dimensional architecture and vascularisation, which is difficult to resolve and trace with two-dimensional imaging techniques^54^. The tree-like villi structures serve to increase the surface area by which products from the maternal blood are made available to the fetus for nutrient intake and studies have shown that branching may be associated with the inverse of the fetoplacental weight ratio, a widely used clinical parameter^55^. Furthermore, villous shape is influenced by vascular development, and abnormal vascular development can be linked to disorders like pre-eclampsia^56,57^. Their undistorted visualisation, especially in 3D and without the use of any contrast agent, is fundamental for extending our understanding of fetal development. The correlation between $\upmu$CT volumes and conventional histology, along with high-resolution XRF elemental maps, suggests that virtual histology can offer comparable or potentially superior information with less stringent requirements and in a non-destructive manner.

In our case, a different sample preparation would have facilitated the tracing of blood vessels (i.e., perfusion fixing techniques^58^). After delivery, the placenta collapses and loses a significant amount of maternal blood, resulting in a sparser distribution of blood cells and deflated vessels. For instance, individual blood cells appear as isolated bright dots in the phase-contrast volume, a finding confirmed by the two-dimensional techniques. Moreover, Fig. 5c shows that tissue damage can occur from prolonged radiation exposure of thin samples during XRF scans.

Both MBI datasets have been acquired at similar experimental conditions and have been reconstructed using the same UMPA algorithm and processing steps. The image quality has been analysed in terms of spatial resolution and CNR. In our measurements we found that the TAI dataset exhibits a 25% higher phase sensitivity than the sandpaper dataset, with shorter acquisition times, while the CNR ratio obtained with the two MBI techniques was comparable. Nevertheless, the advantage of employing a random phase modulator, such as the sandpaper, is the simplicity of the setup. Unlike for the TAIs, the use of the sandpaper does not impose limitations on the geometry of the setup and does not require any specifically microfabricated optical element in the X-ray beam. We found a lower spatial resolution for MBI (about ${8}\,\upmu \hbox {m}$) than for PBI (${5.1}\,\upmu \hbox {m}$), whereas a lower CNR was determined for the PBI dataset. However, it should be noted that both spatial resolution and CNR can be tuned by changing the value of parameters of the phase-retrieval algorithms. For a given dataset of PBI, an increase of CNR normally corresponds to a decrease in spatial resolution, and vice versa. The entire process from image acquisition to data analysis and tomographic reconstruction is in general faster with PBI than with the MBI methods. On the other hand, MBI techniques are able to provide complementary image signals (dark-field, quantitative phase and attenuation information are obtained from the same dataset), which can not be accessed with PBI. In the case of placental tissue, the lack of extended homogeneous regions, where sub-resolution microstructural information could be obtained, limits the detectable small-angle scattering signal. The sample’s structural complexity leads to an increased level of noise in the dark-field signal (see Supplementary Fig. S1). As a result, achieving a reliable dark-field signal becomes particularly difficult, and few dark-field studies have been conducted at this high spatial resolution level using similar methods^28,59^. Furthermore, both the attenuation and dark-field signals are contaminated by propagation fringes, causing the dark-field to primarily emphasize edges rather than the intended signal. With more sophisticated analysis that accounts also for the propagation fringes in the transmission signal, the three signals from MBI could be combined together for, e.g., studies of material decomposition^60,61^. Such studies could provide volumetric information similar to the XRF elemental maps shown in Fig. 5d,f and relative subplots.

Ultimately, the choice of the technique is driven by a number of factors including the sample characteristics, the scientific question addressed in the experiment, and the available experimental facilities. We note that the phase sensitivity and spatial resolution achievable by the PBI and MBI techniques can be tuned depending on the experimental needs, but, at the synchrotron, are ultimately limited to the micrometre range for the spatial resolution, and to angular sensitivities on the order of nanoradians^26–28^. To access even higher spatial resolutions in the near-field regime, other methods could be considered, such as X-ray holotomography^62^ or X-ray near-field ptychography^63^.

While this study demonstrates the potential of phase-contrast $\upmu$CT for virtual histology of human placenta, its application in clinical or routine laboratory settings presents several challenges. Transitioning to laboratory-based X-ray sources would involve significant trade-offs in terms of system stability, resolution, flux, and coherence^25,64^. However, advancements in compact X-ray sources, coupled with phase-contrast imaging setups optimized for laboratory environments, could bridge some of these gaps^65–67^. Another consideration is the optimisation of data acquisition and processing in MBI methods. Simplified setups, continuous acquisition schemes^35,68^ and automated workflows will be essential in making MBI accessible and practical for a broader range of users. Future work could explore integrating AI-driven solutions to expand the accessibility of virtual histology with more efficient scans^69–71^.

## Supplementary Information

Supplementary Information.

## Data Availability

Tomographic data from P05 underlying the results presented in this study may be obtained from the authors upon request, please contact the corresponding author, S.S. XRF data can be found at 10.15151/ESRF-ES-1308373111. The Unified Modulated Pattern Analysis (UMPA) code is available at https://github.com/optimato/UMPA and the drift correction functions at 10.5281/zenodo.8383714.

## References

[1] Fitzgerald, R. Phase-sensitive X-ray imaging. *Phys. Today* **53**, 23–26. 10.1063/1.1292471 (2000).

[2] Momose, A. et al. Demonstration of X-ray talbot interferometry. *Jpn. J. Appl. Phys.* **42**, L866. 10.1143/JJAP.42.L866 (2003).

[3] Pfeiffer, F., Weitkamp, T., Bunk, O. & David, C. Phase retrieval and differential phase-contrast imaging with low-brilliance X-ray sources. *Nat. Phys.* **2**, 258–261. 10.1038/nphys265 (2006).

[4] Olivo, A. & Speller, R. A coded-aperture technique allowing X-ray phase contrast imaging with conventional sources. *Appl. Phys. Lett.* **91**, 074106. 10.1063/1.2772193 (2007).

[5] Bérujon, S., Ziegler, E., Cerbino, R. & Peverini, L. Two-dimensional X-ray beam phase sensing. *Phys. Rev. Lett.* **108**, 158102. 10.1103/physrevlett.108.158102 (2012). 22587288 10.1103/PhysRevLett.108.158102

[6] Morgan, K. S., Paganin, D. M. & Siu, K. K. W. X-ray phase imaging with a paper analyzer. *Appl. Phys. Lett.* **100**, 124102. 10.1063/1.3694918 (2012).

[7] Zanette, I. et al. Speckle-based X-ray phase-contrast and dark-field imaging with a laboratory source. *Phys. Rev. Lett.* **112**, 253903. 10.1103/physrevlett.112.253903 (2014). 25014818 10.1103/PhysRevLett.112.253903

[8] Zdora, M.-C. et al. At-wavelength optics characterisation via X-ray speckle- and grating-based unified modulated pattern analysis. *Opt. Express* **26**, 4989. 10.1364/oe.26.004989 (2018). 29475342 10.1364/OE.26.004989

[9] Di Trapani, V., Brombal, L., De Marco, F., Dreossi, D. & Thibault, P. Unified modulation pattern analysis (umpa) algorithm for 1d sensitive X-ray phase contrast imaging techniques. *J. Instrum.* **18**, C01059. 10.1088/1748-0221/18/01/c01059 (2023).

[10] Morgan, K. S., Paganin, D. M. & Siu, K. K. W. Quantitative single-exposure X-ray phase contrast imaging using a single attenuation grid. *Opt. Express* **19**, 19781. 10.1364/oe.19.019781 (2011). 21996920 10.1364/OE.19.019781

[11] Gustschin, A. et al. High-resolution and sensitivity bi-directional X-ray phase contrast imaging using 2D Talbot array illuminators. *Optica* **8**, 1588. 10.1364/optica.441004 (2021). 37829605 10.1364/OPTICA.441004 PMC10567101

[12] John, D. Recent developments in quantitative phase-contrast microtomography using Talbot array illuminators. In *Developments in X-ray Tomography XV* (eds Müller, B. & Wang, G.) 39 (SPIE, 2024).

[13] Bérujon, S. & Ziegler, E. Near-field speckle-scanning-based x-ray imaging. *Phys. Rev. A* **92**, 837. 10.1103/physreva.92.013837 (2015).

[14] Pavlov, K. M. et al. Single-shot x-ray speckle-based imaging of a single-material object. *Phys. Rev. Appl.* **13**, 23. 10.1103/physrevapplied.13.054023 (2020).

[15] Pavlov, K. M. et al. X-ray multi-modal intrinsic-speckle-tracking. *J. Opt.* **22**, 125604. 10.1088/2040-8986/abc313 (2020).

[16] Alloo, S. J., Morgan, K. S., Paganin, D. M. & Pavlov, K. M. Multimodal intrinsic speckle-tracking (MIST) to extract images of rapidly-varying diffuse X-ray dark-field. *Sci. Rep.* **13**, 1. 10.1038/s41598-023-31574-z (2023). 37012270 10.1038/s41598-023-31574-z PMC10070351

[17] Qiao, Z., Shi, X., Celestre, R. & Assoufid, L. Wavelet-transform-based speckle vector tracking method for X-ray phase imaging. *Opt. Express* **28**, 33053. 10.1364/oe.404606 (2020). 33114975 10.1364/OE.404606

[18] Quénot, L., Rougé-Labriet, H., Bohic, S., Bérujon, S. & Brun, E. Implicit tracking approach for X-ray phase-contrast imaging with a random mask and a conventional system. *Optica* **8**, 1412. 10.1364/optica.434954 (2021).

[19] Rougé-Labriet, H. et al. Comparison of x-ray speckle-based imaging deflection retrieval algorithms for the optimization of radiation dose. *Phys. Med. Biol.* **66**, 065005. 10.1088/1361-6560/ab87f7 (2021). 32268312 10.1088/1361-6560/ab87f7

[20] Zdora, M.-C. et al. X-ray phase-contrast imaging and metrology through Unified Modulated Pattern Analysis. *Phys. Rev. Lett.* **118**, 203903. 10.1103/physrevlett.118.203903 (2017). 28581800 10.1103/PhysRevLett.118.203903

[21] De Marco, F. et al. High-speed processing of X-ray wavefront marking data with the unified modulated pattern analysis (umpa) model. *Opt. Express* **31**, 635–650. 10.1364/OE.474794 (2023). 36606998 10.1364/OE.474794

[22] Paganin, D., Mayo, S. C., Gureyev, T. E., Miller, P. R. & Wilkins, S. W. Simultaneous phase and amplitude extraction from a single defocused image of a homogeneous object. *J. Microsc.* **206**, 33–40. 10.1046/j.1365-2818.2002.01010.x (2002). 12000561 10.1046/j.1365-2818.2002.01010.x

[23] Olivo, A. & Speller, R. Experimental validation of a simple model capable of predicting the phase contrast imaging capabilities of any X-ray imaging system. *Phys. Med. Biol.* **51**, 3015–3030. 10.1088/0031-9155/51/12/001 (2006). 16757859 10.1088/0031-9155/51/12/001

[24] Zdora, M.-C. State of the art of X-ray speckle-based phase-contrast and dark-field imaging. *J. Imaging* **4**, 60. 10.3390/jimaging4050060 (2018).

[25] Quénot, L., Bohic, S. & Brun, E. X-ray phase contrast imaging from synchrotron to conventional sources: A review of the existing techniques for biological applications. *Appl. Sci.* **12**, 9539. 10.3390/app12199539 (2022).

[26] Zdora, M.-C. et al. X-ray phase tomography with near-field speckles for three-dimensional virtual histology. *Optica* **7**, 1221. 10.1364/optica.399421 (2020).

[27] Riedel, M. et al. Comparing x-ray phase-contrast imaging using a Talbot array illuminator to propagation-based imaging for non-homogeneous biomedical samples. *Sci. Rep.* **13**, 7. 10.1038/s41598-023-33788-7 (2023). 37117518 10.1038/s41598-023-33788-7 PMC10144904

[28] Savatović, S. et al. Multi-resolution X-ray phase-contrast and dark-field tomography of human cerebellum with near-field speckles. *Biomed. Opt. Express* **15**, 142–161. 10.1364/BOE.502664 (2024). 38223169 10.1364/BOE.502664 PMC10783905

[29] Laundon, D., Gostling, N. J., Sengers, B. G., Chavatte-Palmer, P. & Lewis, R. M. Placental evolution from a three-dimensional and multiscale structural perspective. *Evolution.*10.1093/evolut/qpad209 (2023). 10.1093/evolut/qpad209 37974468

[30] Lewis, R. M. & Pearson-Farr, J. E. Multiscale three-dimensional imaging of the placenta. *Placenta* **102**, 55–60. 10.1016/j.placenta.2020.01.016 (2020). 33218580 10.1016/j.placenta.2020.01.016

[31] Savatović, S. Extending the field-of-view of speckle-based microtomography with the umpa model. In *Developments in X-ray Tomography XV* (eds Müller, B. & Wang, G.) 31 (SPIE, 2024).

[32] Greving, I. P05 imaging beamline at petra III: First results. In *Developments in X-ray Tomography IX* (ed. Stock, S. R.) (SPIE, 2014).

[33] Wilde, F. et al. Micro-CT at the imaging beamline P05 at PETRA III. In *AIP Conference Proceedings*. 10.1063/1.4952858 (2016).

[34] Lytaev, P. et al. Characterization of the CCD and CMOS cameras for grating-based phase-contrast tomography. In *Developments in X-ray Tomography IX* (ed. Stock, S. R.) (SPIE, 2014).

[35] Savatović, S. et al. Helical sample-stepping for faster speckle-based multi-modal tomography with the Unified Modulated Pattern Analysis (UMPA) model. *J. Instrum.* **18**, C11020. 10.1088/1748-0221/18/11/C11020 (2023).

[36] Bon, P., Monneret, S. & Wattellier, B. Noniterative boundary-artifact-free wavefront reconstruction from its derivatives. *Appl. Opt.* **51**, 5698. 10.1364/AO.51.005698 (2012). 22885583 10.1364/AO.51.005698

[37] van Aarle, W. et al. The ASTRA Toolbox: A platform for advanced algorithm development in electron tomography. *Ultramicroscopy* **157**, 35–47. 10.1016/j.ultramic.2015.05.002 (2015). 26057688 10.1016/j.ultramic.2015.05.002

[38] van Aarle, W. et al. Fast and flexible X-ray tomography using the ASTRA toolbox. *Opt. Express* **24**, 25129. 10.1364/OE.24.025129 (2016). 27828452 10.1364/OE.24.025129

[39] Als-Nielsen, J. & McMorrow, D. *Elements of Modern X-ray Physics* 1 edn. (Wiley, 2011).

[40] Weitkamp, T., Haas, D., Wegrzynek, D. & Rack, A. ANKAphase: Software for single-distance phase retrieval from inline X-ray phase-contrast radiographs. *J. Synchrotron Radiat.* **18**, 617–629. 10.1107/s0909049511002895 (2011). 21685680 10.1107/S0909049511002895

[41] Schoonjans, T. et al. The xraylib library for X-ray–matter interactions. Recent developments. *Spectrochim. Acta B At. Spectrosc.* **66**, 776–784. 10.1016/j.sab.2011.09.011 (2011).

[42] Cotte, M. et al. The ID21 X-ray and infrared microscopy beamline at the ESRF: Status and recent applications to artistic materials. *J. Anal. At. Spectrom.* **32**, 477–493. 10.1039/c6ja00356g (2017).

[43] Gianoncelli, A. et al. Difficulties and artefacts in cryo-fixation of ovarian tissues for X-ray fluorescence analyses. *J. Anal. At. Spectrom.* **38**, 1744–1750. 10.1039/d3ja00164d (2023).

[44] Aloisio, I. A., Paganin, D. M., Wright, C. A. & Morgan, K. S. Exploring experimental parameter choice for rapid speckle-tracking phase-contrast X-ray imaging with a paper analyzer. *J. Synchrotron. Radiat.* **22**, 1279–1288. 10.1107/S1600577515011406 (2015). 26289280 10.1107/S1600577515011406

[45] Zhou, T. et al. Noise analysis of speckle-based X-ray phase-contrast imaging. *Opt. Lett.* **41**, 5490. 10.1364/ol.41.005490 (2016). 27906220 10.1364/OL.41.005490

[46] Van Nieuwenhove, V. et al. Dynamic intensity normalization using eigen flat fields in X-ray imaging. *Opt. Express* **23**, 27975. 10.1364/oe.23.027975 (2015). 26480456 10.1364/OE.23.027975

[47] Salditt, T., Aspelmeier, T. & Aeffner, S. *Biomedical Imaging: Principles of Radiography, Tomography and Medical Physics* (De Gruyter, 2017).

[48] Modregger, P., Lübbert, D., Schäfer, P. & Köhler, R. Spatial resolution in Bragg-magnified X-ray images as determined by Fourier analysis. *Phys. Status Solidi (a)* **204**, 2746–2752. 10.1002/pssa.200675685 (2007).

[49] Mittal, A., Soundararajan, R. & Bovik, A. C. Making a “completely blind’’ image quality analyzer. *IEEE Signal Process. Lett.* **20**, 209–212. 10.1109/lsp.2012.2227726 (2013).

[50] Ruiz-Yaniz, M. et al. Hard X-ray phase-contrast tomography of non-homogeneous specimens: Grating interferometry versus propagation-based imaging. *J. Synchrotron. Radiat.* **23**, 1202–1209. 10.1107/s1600577516009164 (2016). 27577776 10.1107/S1600577516009164

[51] Laundon, D. et al. *Correlative Multiscale MicroCT-SBF-SEM Imaging of Resin-Embedded Tissue, Methods in Cell Biology* 241–267 (Elsevier, 2023). 10.1016/bs.mcb.2023.01.014 37451769

[52] Sadovsky, Y. & Jansson, T. *Placenta and Placental Transport Function* 1741–1782 (Elsevier, 2015).

[53] Belevich, I., Joensuu, M., Kumar, D., Vihinen, H. & Jokitalo, E. Microscopy image browser: A platform for segmentation and analysis of multidimensional datasets. *PLoS Biol.* **14**, e1002340. 10.1371/journal.pbio.1002340 (2016). 26727152 10.1371/journal.pbio.1002340 PMC4699692

[54] Haeussner, E. et al. Does 2D-histologic identification of villous types of human placentas at birth enable sensitive and reliable interpretation of 3D structure? *Placenta* **36**, 1425–1432. 10.1016/j.placenta.2015.10.003 (2015). 26494606 10.1016/j.placenta.2015.10.003

[55] Haeussner, E., Buehlmeyer, A., Schmitz, C., von Koch, F. E. & Frank, H.-G. Novel 3D microscopic analysis of human placental villous trees reveals unexpected significance of branching angles. *Sci. Rep.* **4**, 192. 10.1038/srep06192 (2014). 10.1038/srep06192 PMC4143784 25155961

[56] Hermans, S. et al. Definition and quantification of three-dimensional imaging targets to phenotype pre-eclampsia subtypes: An exploratory study. *Int. J. Mol. Sci.* **24**, 3240. 10.3390/ijms24043240 (2023). 36834652 10.3390/ijms24043240 PMC9959375

[57] Andreuzzi, E. et al. P-424 placental extracellular matrix remodelling in pregnancies by oocyte donation shows similarities with preeclamptic placenta: A pilot study. *Hum. Reprod.* **39**, 774. 10.1093/humrep/deae108.774 (2024).

[58] Tun, W. M. et al. A massively multi-scale approach to characterizing tissue architecture by synchrotron micro-CT applied to the human placenta. *J. R. Soc. Interface* **18**, 20210140. 10.1098/rsif.2021.0140 (2021). 34062108 10.1098/rsif.2021.0140 PMC8169212

[59] Wirtensohn, S. et al. Nanoscale dark-field imaging in full-field transmission X-ray microscopy. *Optica* **11**, 852–859. 10.1364/optica.524812 (2024).

[60] Braig, E.-M. et al. Single spectrum three-material decomposition with grating-based X-ray phase-contrast CT. *Phys. Med. Biol.* **65**, 185011. 10.1088/1361-6560/ab9704 (2020). 32460250 10.1088/1361-6560/ab9704

[61] Brombal, L. et al. Edge-illumination spectral phase-contrast tomography. *Phys. Med. Biol.* **69**, 075027. 10.1088/1361-6560/ad3328 (2024). 10.1088/1361-6560/ad3328 PMC10991267 38471186

[62] Khimchenko, A. et al. Hard X-ray nanoholotomography: Large-scale, label-free, 3D neuroimaging beyond optical limit. *Adv. Sci.* **5**, 1700694. 10.1002/advs.201700694 (2018). 10.1002/advs.201700694 PMC6010902 29938163

[63] Taphorn, K. et al. X-ray stain localization with near-field ptychographic computed tomography. *Adv. Sci.* **9**, 2201723. 10.1002/advs.202201723 (2022). 10.1002/advs.202201723 PMC9404393 35748171

[64] Celestre, R., Quenot, L., Ninham, C., Brun, E. & Fardin, L. *Review of Speckle-Tracking Algorithms for X-ray Phase Contrast Imaging: Low Dose Applications*. 10.48550/arXiv.2404.11633 (2024). 10.1107/S1600577524010117 PMC11708844 39689035

[65] Brombal, L., Arfelli, F., Menk, R. H., Rigon, L. & Brun, F. PEPI lab: A flexible compact multi-modal setup for X-ray phase-contrast and spectral imaging. *Sci. Rep.* **13**, 5. 10.1038/s41598-023-30316-5 (2023). 36918574 10.1038/s41598-023-30316-5 PMC10014955

[66] Di Trapani, V. et al. Speckle-based imaging (SBI) applications with spectral photon counting detectors at the newly established OPTIMATO (OPtimal IMAging and TOmography) laboratory. *J. Instrum.* **19**, C01018. 10.1088/1748-0221/19/01/c01018 (2024).

[67] Zandarco, S. et al. Speckle tracking phase-contrast computed tomography at an inverse compton X-ray source. *Opt. Express* **32**, 28472. 10.1364/oe.528701 (2024). 39538663 10.1364/OE.528701

[68] Lioliou, G. et al. Recent developments in fly scan methods for phase and multi-contrast X-ray micro-CT based on amplitude modulated beams. *Tomogr. Mater. Struct.* **5**, 100034. 10.1016/j.tmater.2024.100034 (2024).

[69] Qiao, Z. et al. Real-time X-ray phase-contrast imaging using SPINNet-a speckle-based phase-contrast imaging neural network. *Optica* **9**, 391. 10.1364/optica.453748 (2022).

[70] Pelt, D. M., Roche i Morgó, O., Maughan Jones, C., Olivo, A. & Hagen, C. K. Cycloidal ct with cnn-based sinogram completion and in-scan generation of training data. *Sci. Rep.* **12**, 1. 10.1038/s41598-022-04910-y (2022). 35042961 10.1038/s41598-022-04910-y PMC8766453

[71] Ge, X. et al. Virtual differential phase-contrast and dark-field imaging of X-ray absorption images via deep learning. *Bioeng. Transl. Med.* **8**, 494. 10.1002/btm2.10494 (2023). 10.1002/btm2.10494 PMC10658538 38023711

