# Supplementary material for: High-resolution X-ray phase-contrast tomography of human placenta with different wavefront markers
Source: Sci Rep. 2025 Jan 16;15:2131. doi: 10.1038/s41598-025-85105-z (PMC11739398; doi:10.1038/s41598-025-85105-z)
Supplement: Supplementary file 1 — Supplementary Information. [file 41598_2025_85105_MOESM1_ESM.pdf]

Supplementary Figures

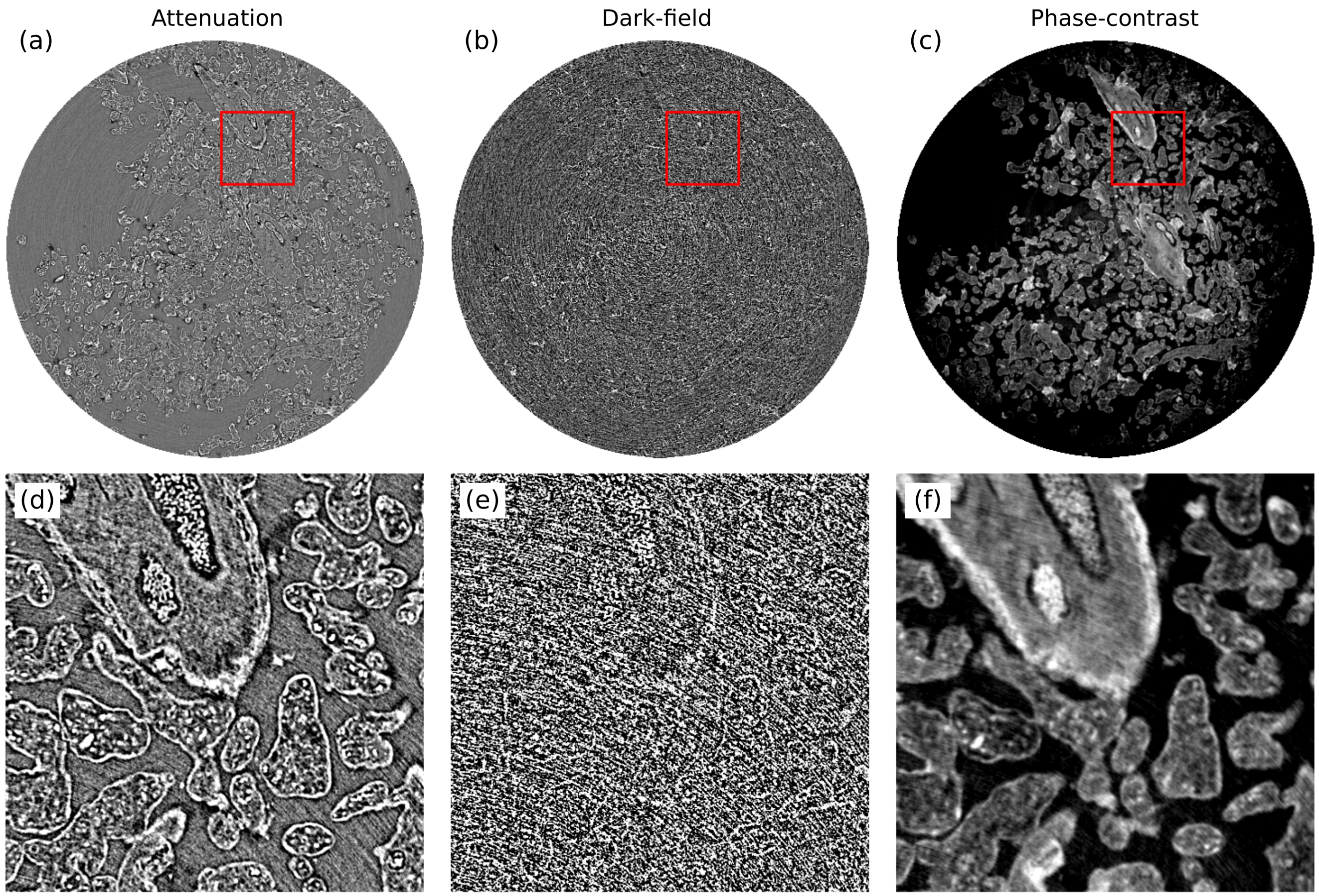

Figure S1. Comparison of the three different signals obtained for MBI with sandpaper. **(a)** Attenuation, **(b)** dark-field and **(c)** phase-contrast reconstructions of the same slice are shown, along with corresponding zoomed ROIs **(d)**-**(f)**. The dark-field signal for this sample was significantly affected by noise, making it challenging to discern any structural details.
